# Supplementary material for: Reduction in malaria burden following the introduction of indoor residual spraying in areas protected by long-lasting insecticidal nets in Western Kenya, 2016–2018
Source: PLoS One. 2022 Apr 20;17(4):e0266736. doi: 10.1371/journal.pone.0266736 (PMC9020686; doi:10.1371/journal.pone.0266736)
Supplement: Attachment — Submitted filename: Response to Reviewers.docx [file pone.0266736.s002.docx]

Dear Editor,

Plos One

12 March 2022

**Ref: Rebuttal Letter**

We thank the editor and the reviewer for their comments on our manuscript. Below are itemized responses to each point raised by the academic editor and reviewer #1. We trust that our responses are adequate and will allow for the manuscript to move to the next step towards publication.

Sincerely,

On behalf of all authors

Diba Dulacha

**Academic Editor**

1. Please ensure that your manuscript meets PLOS ONE's style requirements, including those for file naming:

**We have reviewed the manuscript with a view of ensuring compliance with the style requirements. Files names (figures and tables) have been revised in accordance with the style requirements.**

2. Please provide additional details regarding participant consent. In the ethics statement in the Methods and online submission information, please ensure that you have specified (1) whether consent was informed and (2) what type you obtained (for instance, written or verbal, and if verbal, how it was documented and witnessed).

**This was a retrospective study that involved the review of health facility records. We have clarified that the variables collected are anonymized and no direct identifiers were included. The study has an IRB approval as well as consent from the county government to access the health records.**

**‘Moi University Institution for Research and Ethics Committee (IREC) approved this study (Approval Number: 0002048), and the Health Departments of Migori and Homa Bay Counties gave consent to access the health facilities and the records. The variables collected were fully anonymized monthly summaries from health records. The study underwent human subject review at the U.S. Centers for Disease Control and Prevention and was approved as a program evaluation activity that does not require human subject research review.’**

3. We note that the grant information you provided in the ‘Funding Information’ and ‘Financial Disclosure’ sections do not match.

Our apologies for the lack of clarity. We would like to revise our financial disclosure as follows.

**The study was funded and supported by the Kenya Field Epidemiology and Laboratory Training Program (FELTP), a program within the Kenya Ministry of Health. The program receives funding support from the US PMI and CDC to train field epidemiologists to increase the epidemiologic workforce in the public health system.** **The author(s) received no specific funding for this work.**

**The funders had no role in study design, data collection and analysis, decision to publish, or preparation of the manuscript.**

Funding information

**The study was funded by Kenya Field Epidemiology and Laboratory Training Program (FELTP) (within MoH). The program supported the lead author, who was a field epidemiology resident with the program at the time to conduct the study. The program receives funding support from its partners and the Kenyan Government to train field epidemiologists for the public health system.**

4. Thank you for stating the following financial disclosure: Please state what role the funders took in the study.

The financial disclosure has been revised as below.

**The study was funded and supported by the Kenya Field Epidemiology and Laboratory Training Program (FELTP), a program within the Kenya Ministry of Health. The program receives funding support from the US PMI and CDC to train field epidemiologists to increase the epidemiologic workforce in the public health system. The author(s) received no specific funding for this work.**

**The funders had no role in study design, data collection and analysis, decision to publish, or preparation of the manuscript.**

5. We noted in your submission details that a portion of your manuscript may have been presented or published elsewhere. [This study was part of a thesis project and thesis report is available on Moi University website.] Please clarify whether this [conference proceeding or publication] was peer-reviewed and formally published. If this work was previously peer-reviewed and published, in the cover letter please provide the reason that this work does not constitute dual publication and should be included in the current manuscript.

**The thesis report that is available on Moi University’s website has not been peer-reviewed nor formally published. Besides, the component presented in the report is limited and lacks the advanced statistical analysis that is presented in this paper.**

6. In your Data Availability statement, you have not specified where the minimal data set underlying the results described in your manuscript can be found. PLOS defines a study's minimal data set as the underlying data used to reach the conclusions drawn in the manuscript and any additional data required to replicate the reported study findings in their entirety. All PLOS journals require that the minimal data set be made fully available.

**The minimal data set has been uploaded as Supporting Information. The data is fully anonymized as per the guidelines. There are no ethical or legal restrictions to sharing of the data publicly.**

7. We note that Figure 1 in your submission contain map image which may be copyrighted.

We require you to either (1) present written permission from the copyright holder to publish these figures specifically under the CC BY 4.0 license, or (2) remove the figures from your submission:

**Please note that Figure 1 (now renamed S1 Fig) is not copyrighted. This figure has been prepared for this manuscript.**

8. Please review your reference list to ensure that it is complete and correct.

**The references have been reviewed as advised and no issues were noted at this point.**

9. Additional Editor Comments: This is a good manuscript that will be acceptable for publication once the authors address the minor revisions raised by the reviewer.

**Thank you for the review and comments. We have revised the manuscript as per the comments by the academic editor and reviewer #1. We trust that the manuscript is now in compliance with the submission and publications requirements and will be considered for the subsequent steps towards publications.**

**Reviewer #1**

1. Is the manuscript technically sound, and do the data support the conclusions?

The manuscript must describe a technically sound piece of scientific research with data that supports the conclusions. Experiments must have been conducted rigorously, with appropriate controls, replication, and sample sizes. The conclusions must be drawn appropriately based on the data presented.

Reviewer #1: Yes

**No revision is required**.

2. Has the statistical analysis been performed appropriately and rigorously?

Reviewer #1: Yes

**No revision is required**.

3. Have the authors made all data underlying the findings in their manuscript fully available?

Reviewer #1: No

**The minimal data set has been uploaded as Supporting Information. The data is fully anonymized as per the guidelines. There are no ethical or legal restrictions to sharing of the data publicly.**

4. Is the manuscript presented in an intelligible fashion and written in standard English?

Reviewer #1: Yes

**No revision is required**.

5. Review Comments to the Author: Please use the space provided to explain your answers to the questions above. You may also include additional comments for the author, including concerns about dual publication, research ethics, or publication ethics. (Please upload your review as an attachment if it exceeds 20,000 characters)
   a. This study is based on data from two sub-counties, Nyatike, and 49 Suba, located on the shore of Lake Victoria, Western Kenya in adjacent counties, Migori and Homa 95 Bay, where there 96 is intense malaria transmission throughout the year. The interventions reported on were conducted from February 1, 2016 to 50 March 31, 2018.

--Please add a comment on how representative the data from Nyatike and Suba are of data from this subregion.

Comment added to demonstrate the similarity between the two sub-counties and their representativeness of the region.

**Both areas are malaria-endemic, had moderate rates of LLIN possession, experienced similar climatic and rainfall patterns and are representative of the Lakes endemic counties.**

b. The authors state that “Kenya conducted three free mass net distribution campaigns in 2011–2012, 2014–2015, and 2017–2018”.

--Details of the date(s) and organisation(s) responsible for net distribution should be stated for the sub-counties that provided the data for this article.

**The net distribution campaigns were conducted in the Lake malaria-endemic counties in June 2011, June 2014 and June 2017 by the National Malaria Control Program with support from Global Fund, US President’s Malaria Initiative (PMI) and other partners.**

c. IRS was conducted in February – March 2017 in Nyatike but not in Suba. One year later both Nyatike and Suba sub-counties received IRS.

--Please add details of the organisation responsible for IRS for these two sub-counties and the reason for some counties receiving IRS and not others.

Please the revision include in the manuscript.

**The IRS campaigns were implemented by the National Malaria Control Program with support from the US President’s Malaria Initiative (PMI).**

d. Two health facilities, one from Nyatike and one from Suba sub-county, provided out-patient data from February 1, 2016, to March 31, 2018. The data included outpatient department attendance, suspected malaria cases, those tested for malaria and those testing positive for malaria.

--was the data collection blinded?

--was it double entered?

**The data collection was not blinded as this was not practical. We used local data collectors who are informed about the implementation of the vector control measures.**

**The data were double entered into Excel before being cleaned and transferred to STATA for analysis.**

e. lines 177 -180. “..entered data into MS Excel for cleaning”. Excel is a spreadsheet application and not a data management programme. Was the data double entered?

**The data was double entered and transported to STATA for analysis.**

f. 6. Line 187. “Months with missing values were imputed”. Please add the frequency that months were missing.

**The missing records for the number of malaria tests performed and the number of confirmed malaria cases for February 2016, February 2017, and December 2017 were computed for the comparison area**

g. 7. The authors have omitted line numbers from Table 1 onwards.

**Line numbering has been included for the entire document.**

h. 8. Results are clearly presented.

**Thank you; no revision is required**.

i. 9. The discussion could be more succinct

**A few revisions were done in tracked changes**.

6. PLOS authors have the option to publish the peer review history of their article (what does this mean?). If published, this will include your full peer review and any attached files.

**Yes.**
